# Post-traumatic Bone Granuloma Caused by Retained Foreign Bodies

**DOI:** 10.7759/cureus.6959

**Published:** 2020-02-11

**Authors:** Riccardo De Angelis, Ruth Duttmann, Paolo Simoni

**Affiliations:** 1 Diagnostic Imaging, Institut Bordet, Brussels, BEL; 2 Anatomopathology, Centre Hospitalier Universitaire Brugmann, Brussels, BEL; 3 Radiology and Medical Imaging, Queen Fabiola Children's University Hospital, Brussels, BEL

**Keywords:** bone granuloma, foreign body, pseudotumors

## Abstract

Bone granulomas (BGs) due to foreign bodies are a rare condition, especially in children, with only few cases reported in literature. As foreign bodies are not always visible on imaging, BGs can mimic bone tumors. We hereby present a case of a six-year-old boy with histopathologically confirmed BG of his right hand fifth finger due to intraosseous foreign bodies, along with imaging work-up.

## Introduction

Foreign body granulomas in soft tissues are well-known entities, both in adults and children [1-2]. On the other side, bone granulomas (BGs) originating from foreign bodies are a rare condition, especially in children, with very few cases reported in literature [3-5]. BGs may either originate from an inflammatory reaction to intraosseous foreign bodies or from contiguity to a soft tissues foreign body granuloma. BG usually presents as an osteolytic lesion with adjacent soft tissues swelling, often with cortical interruption [6]. These radiological findings, together with the difficulty in identifying a foreign body, could raise the suspicion of a bone tumor. Biopsy should be performed in uncertain cases to confirm the diagnosis [7]. In children, the anamnesis of a previous penetrating trauma can be challenging. We present a case of a six-year-old boy presenting with a one-month history of swollen and painful hand/finger; the radiological and histopathological findings supported the clinicians in a rare diagnosis in childhood, the foreign body related BG.

## Case presentation

A six-year-old boy was referred to the ED because of pain and soft tissue swelling of fifth finger in his right hand which was been increasing for one month. The patient presented no impairment of functionality of the finger and no purulent reaction. In the clinical records, there was a superficial soft tissue trauma one month earlier, as the child fell while playing in the garden. 

At the ED admission, the patient had no fever and no sign of inflammatory syndrome from laboratory tests. 

**Figure 1:**
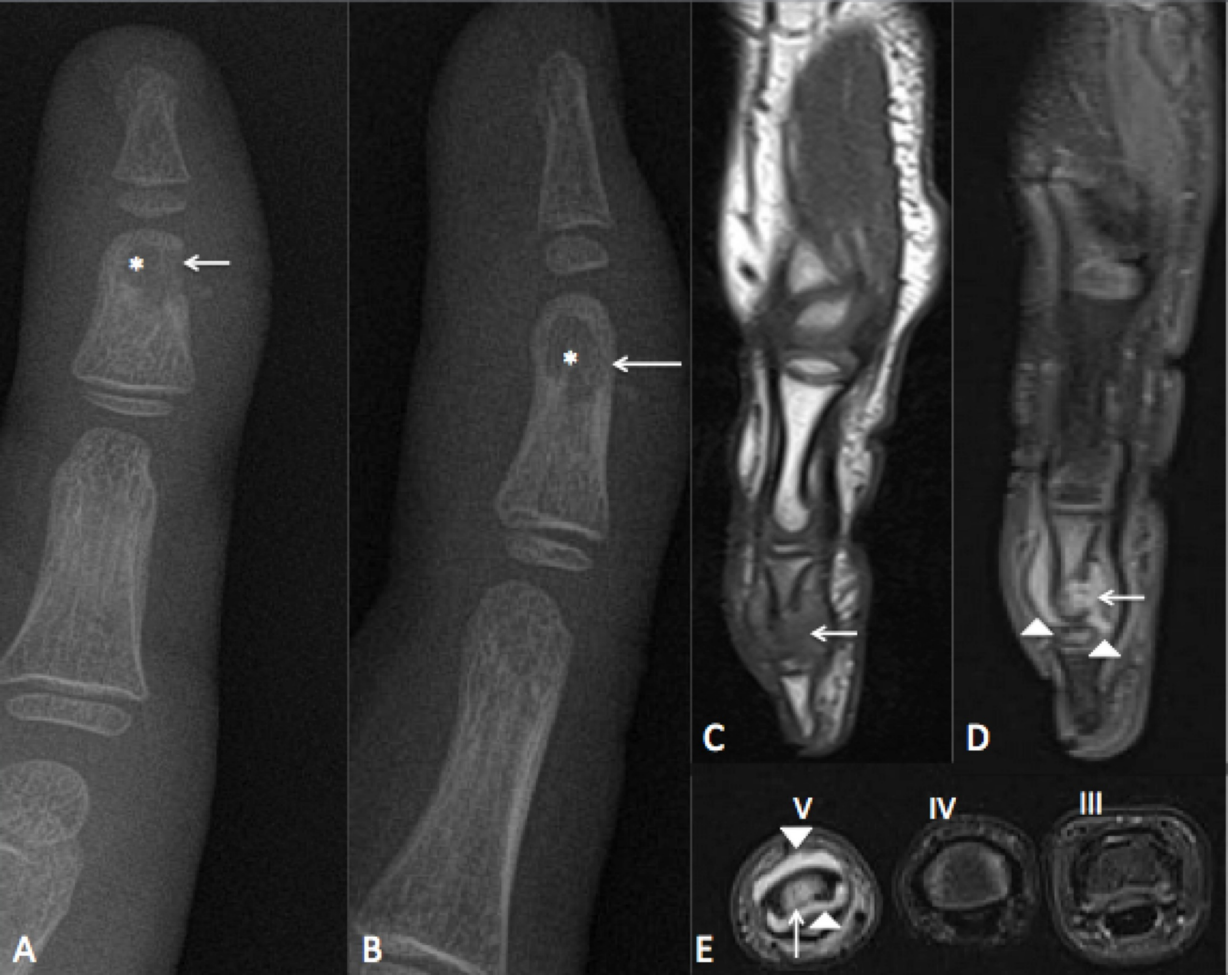
Radiographs and MRI findings. A) Frontal view radiograph of the fifth finger showing a round radiolucent lesion (*) with cortical interruption (arrow) and swelling of surrounding soft tissues; B) Lateral view radiograph showing the same findings; C) Sagittal T1-weighted images showing a low-signal bone marrow replacement lesion with cortical interruption of palmar aspect of finger (arrows); D) Sagittal T2-weighted fat-sat images showing a high signal bone marrow edema due to granulomatous reaction with cortical defect (arrow). The inflammation extended to surrounding soft tissues and to interphalangeal joint (arrowheads); E) T2 weighted fat-sat images on third (III), fourth (IV), and fifth (V) fingers. High-signal bone edema, cortical defect (arrow), and inflammation extension to surrounding soft tissues (arrowheads) can be seen.

Radiographs showed a round-shaped radiolucent lesion of the middle phalanx of the right hand fifth finger with a volar interruption of the cortex. No periosteal reaction was visible. The adjacent soft tissues were swollen. The joint space appeared normal (Figure 1) .

In correspondence of the bone lesion at radiographs, the MRI revealed a replacement lesion in hypo-signal in T1-weighted sequences and hyper-signal on T2-weighted images. Besides, MRI showed an extension to the nearby soft tissues around the phalanx to the distal interphalangeal joint (Figure 1). 

**Figure 2:**
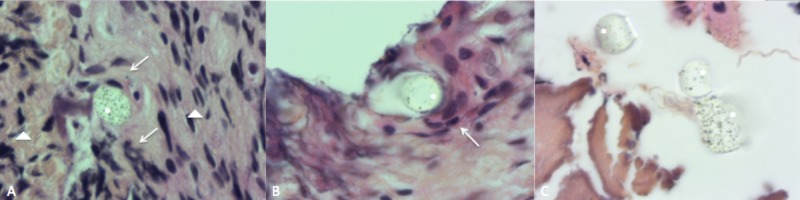
Biopsy pathological specimens. A) Pathological specimens showing microscopic foreign bodies (*) surrounded by multinucleate giant cells and macrophages (arrows). In the periphery we can see amass of lymphocytes and fibroblasts (arrowhead). B) Another foreign body (*) surrounded by  giant cells and macrophages (arrows). C) Foreign bodies (*) with near non vital bone fragments (arrowheads).

A surgical biopsy was performed to establish the nature of the mass because of its nonspecific appearance on imaging. Pathological specimens showed necrotized and fragmented woven bone surrounded by inflammatory cells. No bacteria were evident at the Gram staining. A culture was performed and resulted negative. Instead, some microscopic foreign bodies were identified in the center at the proximity of multinucleate giant cells (MGCs) and macrophages. The foreign bodies appeared as synthetic fibers but their exact origin was unclear (Figure 2).

The final diagnosis consisted of a BG due to intraosseous foreign bodies. After surgical curettage, and oral antibiotic treatment (flucloxacilline), the patient showed a good clinical evolution at three weeks follow-up. Three months after the surgery, the patient lasted asymptomatic with a complete disappearance of the soft tissue swelling. No follow-up radiographs were performed.

## Discussion

Bone granulomas caused by foreign bodies in children are rare, with only a few cases described in the literature [3-5]. It was suggested that BGs originate from extension of the granulomatous tissue of the contiguous soft tissues [6]. In our case, we observed a focal cortical effraction with a continuity inflammatory reaction to near soft tissues and interphalangeal joint. Foreign body may be difficult to find for a long time, especially in children, leading to complications [2]. One must bear in mind that low density or small size foreign bodies can be overlooked on radiographs [7]. Ultrasound (US) may also not identify small foreign bodies and cannot properly investigate bone structures [8]. In these cases, MRI allows tapering the differential diagnosis, mainly when small size foreign bodies are found into the mass due to their ferromagnetic content [7]. In our case, the foreign body was not visible due to its nature and small size on radiographs. US was not performed because foreign body presence was not suspected. We performed then an MRI to evaluate the bone lesion seen on radiographs and the surrounding tissues. The extremity foreign body in pediatric age group has its own set of characteristics and differential diagnosis. As in the presented case, BG can simulate other bone lesions typical of the phalanges, including enchondroma, nonossifying fibroma (NOF), osteoid osteoma, solitary, and aneurysmal bone cyst or fibrous cortical defect. In our case, enchondroma was excluded due to the age of patient and location of the lesion [9]. Osteoid osteoma was not considered because it presents a typical appearance of intralesional nidus and surrounding sclerosis, and it is commonly found in patients between the ages of 10 and 35 [10]. Solitary cysts are often discovered right after trauma as pathological fractures and are most common in proximal humerus and femur [11]. In aneurismal cysts we usually find intralesionals trabeculae and fluid-fluid levels [12]. Fibrous cortical defects have similar radiological aspect to our case, but are very rarely seen in upper limbs [13]. Finally, NOF are often asymptomatic and they are surrounded by osteoblastic reaction [13]. In our case it was not possible to characterize the lesion with imaging techniques, so the final diagnosis required a surgical biopsy and the identification of foreign bodies. When the foreign bodies are not visible, a surgical curettage and biopsy are mandatory to establish its nature. Surgical removal of foreign bodies with associated antibiotherapy is the treatment of choice [1, 7]. Our patient showed a rapid response to treatment and just few months after the surgery all symptoms disappeared, with no long-term consequences.

## Conclusions

Bone granulomas caused by an intraosseous foreign body is a rare condition in children. BG should be included in the differential diagnosis of lytic lesion of the hand phalanges in children, even in case of nonpenetrating trauma. 
